# Parental anxiety and depression are associated with adverse mental health in children with special needs during the COVID-19 pandemic

**DOI:** 10.3389/fpubh.2023.1254277

**Published:** 2023-11-21

**Authors:** Piotr Gruszka, Kristin Ganahl, Nicole Stasch, Christoph Burger, Edda Haberlandt, Susanne M. Bauer

**Affiliations:** ^1^Agency for Preventive and Social Medicine, Bregenz, Austria; ^2^Department of Developmental and Educational Psychology, Faculty of Psychology, University of Vienna, Vienna, Austria; ^3^Department of Cognition, Emotion, and Methods in Psychology, Faculty of Psychology, University of Vienna, Vienna, Austria; ^4^Department of Psychology and Psychodynamics, Karl Landsteiner University for Health Sciences, Krems, Austria; ^5^Department of Pediatrics, Hospital Dornbirn, Dornbirn, Austria

**Keywords:** mental health, parents, children, depression, anxiety, COVID-19, special needs

## Abstract

**Introduction:**

The coronavirus disease 2019 (COVID-19) pandemic has led to restrictions in various areas of life, including social life, work, leisure, health, and education. Vulnerable groups, such as children with special needs and their parents, may be at increased risk of experiencing exacerbated mental health problems during stressful periods such as the COVID-19 lockdowns.

**Materials and methods:**

Telephone interviews were conducted with 954 parents of children with special needs. We assessed parental levels of generalized anxiety and depression using the validated GAD-7 and PHQ-8 scales. Parents were asked to rate family burden and their worry about the COVID-19 crisis, as well as their children's adverse mental health symptoms and health behaviors. Parents also reported their children's worries about the COVID-19 crisis. We conducted regressions to examine the relationship between parents' mental health problems and their children's adverse mental health symptoms and health behaviors. Qualitative data from open-ended questions were coded thematically and major themes of parental worry about the COVID-19 crisis were identified.

**Results:**

Parental anxiety and depression symptoms predicted adverse mental health symptoms and behaviors in children with special needs. Criteria for current depression were met by 7.9% of parents of children with special needs, whereas 4.7% of the general population in Vorarlberg met the criteria for current depression according to data from the Austrian Health Interview Survey in 2019. Parental self-ratings of both depression and anxiety were highly correlated. The majority of parents reported being burdened (79.1%) or worried (67.8%) about the COVID-19 crisis. The main themes of parental worry about the COVID-19 crisis included COVID-19 infection (40.6%), economic situation (13.1%), uncertainty (8.4%), lack of social contact with family and friends (8.1%), family health status (7.5%), and school life (7.5%).

**Discussion:**

Mental health symptoms in parents of children with special needs were strongly associated with increased adverse mental health symptoms and health behaviors in their children. Parents of children with special needs were more likely to be depressed during the COVID-19 pandemic than adults in 2019. We call for additional mental health support to reduce the mental health burden in families with children with special needs.

## Introduction

The COVID-19 pandemic was an international public health emergency that had an enormous impact on the daily lives and routines of people around the world. Countries and territories imposed lockdowns to prevent the spread of the severe acute respiratory syndrome coronavirus 2 (SARS-CoV-2), resulting in restrictions in several areas of life, including social life, work, leisure activities, health, and education. Meta-analyses of longitudinal studies found that healthy individuals were generally resilient to the effects of these restrictions, but showed considerable heterogeneity between studies and for different subgroups (1, 2), with some groups being more vulnerable than others. Vulnerable groups such as children with special needs and their parents may be at particularly high risk of experiencing exacerbated mental health problems during highly distressing periods such as the COVID-19 pandemic and lockdowns. However, only very few studies have been conducted on the mental health of children and adolescents with special needs and their parents during the COVID-19 pandemic. Furthermore, most mental health studies related to COVID-19 have been limited by methodological limitations. The present study aims to overcome these limitations and aims to extend the literature by examining the association between parents' mental health problems and their children's mental health symptoms during the COVID-19 pandemic.

### Adverse effects of the COVID-19 pandemic on children and adolescents

Children are particularly vulnerable because they are highly dependent on others to meet their basic needs (3). A substantial number of studies have demonstrated adverse effects of the COVID-19 pandemic on mental health symptoms, health behaviors and wellbeing in children and adolescents (4–8). In a narrative synthesis, Viner et al. (9) showed that school closures as part of broader social lockdowns during the first wave of COVID-19 were associated with adverse mental health symptoms (such as anxiety and distress) and adverse health behaviors (such as increased screen time and decreased physical activity) in children and adolescents. Another recent systematic review found that children felt more anxious, depressed, fatigued, and distressed during the COVID-19 pandemic (10). A recent meta-analysis (11) also found elevated symptom levels of depression and anxiety symptoms in mothers of young children (under 5 years of age).

### The situation of children with special needs

Children with special needs (i.e., neurodevelopmental, mental, genetic, internal, and orthopedic disorders, and developmental disabilities) are an even more vulnerable subgroup and may have been at even greater risk of mental health problems during the COVID-19 pandemic. Unfortunately, very few studies have been conducted on children with special needs and their families during this time (12). A study in Germany showed that vulnerable children and adolescents were at higher risk of experiencing poor health-related quality of life, mental health problems, anxiety symptoms, and depressive symptoms (4). Furthermore, children and adolescents with neurodevelopmental disorders, such as autism spectrum disorder (ASD) and attention-deficit/hyperactivity disorder (ADHD), had a higher prevalence of emotional symptoms and conduct problems, and exhibited fewer prosocial behaviors than neurotypical controls during the COVID-19 pandemic (13). In addition, all groups (ASD, ADHD, and controls) showed exacerbated emotional symptoms compared to pre-COVID-19 levels. Some studies showed that anxiety levels decreased for some children with special needs as the lockdown progressed and schools reopened, but this was not the case for young people with autism (14, 15).

### The situation of parents of children with special needs

Another group of vulnerable individuals who may be at increased risk of mental health problems during the pandemic are parents of children with special needs. Due to a lack of research, their situation during the pandemic remains largely unexplored. In general, parents of children with special needs experience elevated levels of parental stress (16–19) and face a variety of stressful life events, such as the various iterations of their children's diagnostic process (20). Many studies have shown that they are more likely to meet criteria for depression and anxiety disorders (21–26). They also face increased direct economic costs, such as the financial cost of therapy, and indirect economic costs, such as reduced paid hours, missed opportunities for career advancement or time away from paid work in order to accompany the child to treatment (27). There may also be costs such as loss of friends due to lack of time and tension between parents (28). Parents of children with mental disorders may also experience stigma, blame, fear, and exhaustion due to day-to-day demands they face (29, 30).

According to the buffering hypothesis, social support protects (“buffers”) people from the negative consequences of stressful events (31). Consistent with this hypothesis, having access to resources and social support has been shown to have a significant positive impact on the mental wellbeing of parents of children with special needs (32). Lack of social support, on the other hand, can lead to parental depression and anxiety symptoms (33). As pandemic-related social restrictions led to social isolation and restricted social support, this may have had a negative impact on mental health (34). Social restrictions may have had a particularly detrimental effect on parents of children with special needs, as social isolation may have completely undermined the buffering effect of social support. This hypothesis is consistent with the findings of a scoping review, which showed that parents of children with autism spectrum disorder experienced increased demands, stress, and mental health-related symptoms during lockdowns. It also suggested potential long-term effects of prolonged exposure to increased demands on the mental health and wellbeing of parents and families of individuals with autism spectrum disorder (35). Another study showed that lack of social support, parental stress, and parental mental and behavioral problems were associated with elevated levels of state anxiety among parents of children with special needs during the COVID-19 crisis (36).

### The association between parental and child psychopathology

There is a strong relationship between parental and child psychopathology (37–40). Several studies have shown that parental depression is strongly related to offspring psychopathology (39, 41, 42). In addition to genetic factors, several environmental factors have been implicated in this relationship. Negative cognitive styles of depressed parents (43), adverse parenting (44), relationship quality between parents (45), and high levels of stress (38, 44) have been implicated in the transmission of depressive symptoms from parents to children (38). This link between parental and child psychopathology may be even more pronounced for highly vulnerable groups, such as children with special needs and their parents, during highly stressful periods, such as the COVID-19 pandemic.

### Overcoming methodological issues of previous studies

In addition to a lack of studies on highly vulnerable groups such as children with special needs and their families (12), previous studies related to COVID-19 have been limited by several methodological issues, such as self-selected/self-reporting participants, small sample sizes, virtual-only data, heterogeneous samples, and short-term outcomes (46). Online questionnaires, which were the most preferred survey mode for mental health studies during the COVID-19 crisis, can also considerably limit data quality (47). In general, online questionnaires tend to have lower response rates (48), and it has been shown that the longer the questionnaire, the less likely respondents are to start and complete it. Responses to questions placed in later parts of these questionnaires tend to be faster, shorter, and more uniform, which are all characteristics associated with lower quality data (49). Online surveys also lack real-time interaction and personalisation of face-to-face interviews. Unfortunately, large-scale surveys using face-to-face interviews are usually not feasible because they are too expensive, time consuming and logistically burdensome due to travel requirements. Telephone interviews, on the other hand, combine real-time interaction (e.g., opportunity to clarify misunderstandings) and cost-effectiveness (50), and are considered a balanced compromise for many large-scale population surveys, such as the German health update (GEDA) in Germany (51) or the Austrian Health Interview Survey (ATHIS) in Austria (52). However, compared to face-to-face interviews, during telephone interviews it may be more difficult to establish rapport due to the lack of visual cues such as facial expressions or gestures, which may lead to different responses on the phone than in person (50, 53).

### Aims of the present study

The present study investigated whether there is an association between parents' mental health problems (anxiety and depression), family burden and their children's mental health symptoms and behaviors (increased aggression, media use, and sleep problems) during the COVID-19 pandemic in Vorarlberg, a region of Austria, Europe. A large sample of parents of children with special needs was recruited and interviewed by telephone. We ensured high quality data by overcoming major methodological limitations of previous studies, such as online data collection, self-selection bias, random answering, low response rates, as well as small and heterogeneous samples. Parents responded to questions measuring their family burden, their concerns/worries about the COVID-19 crisis, their generalized anxiety and depression symptoms, and their children's adverse mental health symptoms and health behaviors. To gain a better understanding of parents' worry about the COVID-19 crisis, the study also aimed to identify major underlying themes. We hypothesized that increased parental symptoms of generalized anxiety and depression would predict adverse mental health symptoms and health behaviors in children with special needs.

## Materials and methods

### Study design

The study was designed as a cross-sectional telephone survey conducted shortly after the first COVID-19 lockdown in Austria which took place from March 16 to May 1, 2020. The survey consisted of closed-ended and open-ended questions. Trained staff conducted semi-structured telephone interviews with parents (or guardians) of children (or adolescents) with special needs (neurodevelopmental, mental, genetic, internal and orthopedic disorders, and developmental disabilities) who received therapeutic services from aks Kinderdienste. Aks Kinderdienste is an institute in Vorarlberg, Austria, which provides a variety of therapies (e.g., speech therapy, occupational therapy, physical therapy, psychological therapy, and music therapy) for children and adolescents with special needs. All interviews were conducted between May 19 and June 29, 2020. The average interview lasted ~30 min. Before asking the survey questions, interviewers explained the purpose and objectives of the interview, privacy measures, as well as voluntary participation. Participants were given the opportunity to ask questions before giving their verbal consent. The interviewers documented the responses with paper and pencil; in a second step, the responses were entered into a digital data entry sheet. To ensure data quality, every tenth data entry was double-checked. Qualitative data from responses to open-ended questions were categorized independently by two researchers (KG, NS) following a consensus meeting. The results of the final coding were discussed, and the final common themes were agreed upon by the researchers. Parents provided both self-reports and proxy reports about their child.

### Participant characteristics

A total of 954 parents or guardians (51.2%^1^ of those approached) participated in the telephone interview. Their age ranged from 16 to 73 years (*M* = 37.7; *SD* = 7.0). The age of their children ranged from <1 to 19 years (*M* = 6.5; *SD* = 3.9). Further Participant characteristics of the interviewed parents and their children are shown in Tables 1, 2.

**Table 1:** Parent characteristics.

**Parent characteristics**	**Frequency**	**Percentage**
**Gender**		
Female	834	87.9
Male	115	12.1
**Relation to child**		
Mother	814	85.8
Father	95	10.0
Stepmother/stepfather	6	0.6
Other	7	0.7
**Language preference**		
German	730	76.9
Turkish	89	9.4
Other	93	9.8

**Table 2:** Child characteristics.

**Child characteristics**	**Frequency**	**Percentage**
**Age**		
Up to 3 years	190	21.0
4–6 years	336	37.2
7–10 years	229	25.4
11–19 years	148	16.4
**Gender**		
Female	622	65.5
Male	327	34.5

### Measures and covariates

#### Sociodemographic variables

Parents answered interview questions about their sociodemographic status. They were asked about their age, gender, type of relation to the child, employment status, place of work (e.g., office, home etc.), preferred language, place of birth, marital status, highest level of education attained, number of children in the household, childcare, and COVID-19 risk group status. Parents also answered questions about their child's age, gender, and place of birth.

#### Parents' depression and generalized anxiety

##### Parents' depression

Parental depression was assessed using the eight-item Patient Health Questionnaire Depression Scale (PHQ-8). The PHQ-8 is a valid diagnostic and severity measure of depressive disorders. It uses a four-point Likert scale with the response options *not at all* (0), *several days* (1), *more than half the days* (2), and *nearly every day* (3), which are summed to form a total score between 0 and 24 (55). Cut-off points of 5, 10, and 15 are interpreted as representing *minimal* (0–4), *mild* (5–9), *moderate* (10–14), and *severe* (15–24) depression, respectively.

##### Parents' generalized anxiety

Parental anxiety was assessed using the seven-item Generalized Anxiety Disorder Scale (GAD-7). The GAD-7 is a validated diagnostic measure that uses a four-point Likert scale with response options *not at all* (0), *several days* (1), *more than half the days* (2), and *nearly every day* (3), which are summed to create a total score between 0 and 21. Cut-off points of 5, 10, and 15 are interpreted as representing *minimal* (0–4), *mild* (5–9), *moderate* (10–14), and *severe* (15–21) anxiety, respectively (56).

#### Family burden and worry due to the COVID-19 crisis

New items were developed to measure family burden and worry due to COVID-19.

##### Family burden due to the COVID-19 crisis

Family burden due to COVID-19 was assessed by the question “How much of a burden has the COVID-19 crisis been on your family overall?” with the three response options *not a burden, slightly burdensome*, and *very burdensome*.

##### Parents' and children's worry about the COVID-19 crisis

Parental worry about COVID-19 was assessed by the question “Are you or have you been worried due to the COVID-19 crisis?” with the three response options *not at all, somewhat* and *very*. Similarly, children's worries were assessed with the question “Has your child been worried about the COVID crisis?” with the three response options *not at all, somewhat* and *very*.

##### Parental worry themes during the COVID-19 crisis

Major themes of parental worry about COVID-19 were assessed with the open-ended question “What has been your greatest worry during the COVID crisis?”. The fear of their child contracting COVID-19 was assessed with the question “Have you been afraid that your child might contract COVID?” with the dichotomous response options *yes* and *no*. The fear of their child dying from COVID-19 was measured by the question “Have you been afraid that your child might die due to COVID-19?” with the dichotomous response options *yes* and *no*.

#### Children's adverse mental health symptoms and health behaviors

Questions for parents to assess their child's media use, problematic sleep patterns, aggression and impulsivity, and activity were based on two previous studies in German-speaking countries (57, 58).

##### Children's activity

Parents rated their children's activity using two questions: “Thinking about a typical week, on how many days is your child active for at least 60 min (e.g., riding a bike, walking, climbing stairs, dancing, and playing soccer)?” with the response options *0 days, 1 day, 2 days, 3 days, 4 days, 5 days, 6 days*, and *7 days* and “Overall, was your child more or less active during the COVID crisis?” with the response options *more active, less active, about the same* and *don't know*.

##### Children's aggression and impulsivity

Parents rated their children's aggression und impulsivity by answering two questions: “Is your child prone to aggression and impulsive behavior?” and “Has the aggressive behavior of your child increased during the COVID crisis?”. Both questions were answered on a dichotomous scale with the response options *yes* and *no*.

##### Children's media use

Parents rated their children's media use by answering the following questions: “On an average day, how much time does your child spend using a smartphone or tablet?”, “On an average day, how much time does your child spend watching television?”, “On an average day, how much time does your child spend playing computer games?”, and “On an average day, how much time does your child spend interacting (in person or online/by phone) with other friends?”. Response options were *none*, <*10 min, between 11 and 60 min, 1–2 h, 2–3 h, 3–4 h, 4–5 h, 5–6 h, more than 6 h*, and *don't know*. In addition, the increase in media use was assessed by the question “Overall, has your child spent more time using media (smartphone, tablet, computer games) during the COVID crisis?” with the response options *yes, no* and *don't know*.

##### Children's sleep problems

Parents rated their children's sleep problems by answering the following questions: “Does your child have difficulty falling asleep?”, “Has your child's difficulty falling asleep increased since the COVID crisis?”, and “Has your child's sleep pattern become disturbed during the COVID crisis?” with the response options *yes* and *no*.

##### Children's daily routine

Difficulty in establishing a new daily routine during the COVID-19 crisis was assessed by the question “Has it been difficult for your child to establish a new daily routine (e.g., brushing the teeth in the morning)?” with the response options *very difficult, rather difficult, rather easy*, and *very easy*.

### Analytic strategy

As a first step, parents' sociodemographic data, parents' generalized anxiety and depressive symptoms, family burden, worry about the COVID-19 crisis, and their children's symptoms (activity, aggression/impulsivity, media use, and sleep problems) were examined using descriptive statistics (frequencies, means, and standard deviations). Symptom severity of GAD-7 and PHQ-8 scores was categorized as *minimal, mild, moderate*, and *severe* according to Kroenke et al. (55) and Spitzer et al. (56). In a next step, Pearson correlations (*r*) were conducted for continuous variables and biserial correlations (*rb*) for dichotomous variables (with an underlying continuum). Finally, a series of logistic regressions were calculated to examine the relationship between parents' generalized anxiety and depression (predictor variable) and their children's symptoms of increased aggression, media use and sleep problems (outcome variables). All analyses were conducted in R version 4.1.2.

## Results

### Parental generalized anxiety and depressive symptoms

A total of 7.3% and 7.9% of parents met the cut-off of 10 on the GAD-7 (*M* = 2.86, *SD* = 3.78) and the PHQ-8 (*M* = 2.85, *SD* = 3.84) scales, respectively, corresponding to a diagnosis of generalized anxiety disorder and depressive disorder. The severity of the parents' anxiety and depressive symptoms is shown in Table 3. GAD-7 and PHQ-8 scores were significantly and highly positively correlated (*r* = 0.78, *p* < 0.001).

**Table 3:** Severity of parents' anxiety and depressive symptoms.

	**Parental generalized anxiety (GAD-7)**		**Depression (PHQ-8)**	
**Symptom severity**	**Frequency**	**Percentage**	**Frequency**	**Percentage**
Minimal	674	76.94	675	78.13
Mild	138	15.75	121	14.00
Moderate	46	5.25	53	6.13
Severe	18	2.05	15	1.74

### Family burden and parents' worry due to the COVID-19 crisis

The majority of parents (79.1%) reported that their family was slightly (51.7%) or very (27.4%) burdened due to the COVID-19 crisis, while about one-fifth of parents (20.8%) responded that they were not burdened at all. Most parents (67.8%) reported being somewhat (47.5%) or very (20.3%) worried about the COVID-19 crisis, while about one-third of parents (32.2%) reported not being worried at all. According to their parents, the large majority of children (73.1%) were not at all worried about the COVID-19 crisis, while about one-fifth (22.1%) of children were somewhat worried, and only a small minority (4.8%) were very worried. Even the majority of older children (63.3%) aged 10 years or older were not at all worried according to their parents. Approximately one-third (32.1%) of parents worried that their child would contract COVID-19, and 14.6% worried that their child could even die from COVID-19. More than one-third (37.1%) of parents reported worrying about another wave of COVID-19, while about two-thirds (62.9%) reported not worrying at all.

### Major themes of parental worry about the COVID-19 crisis

Major themes of parents' worry during the COVID-19 crisis are presented in Table 4. Most parents worried about themes related to a COVID-19 infection (40.6%).

**Table 4:** Anxiety themes of parents during the COVID-19 crisis.

**Category**	**Frequency**	**Percentage**
COVID infection	276	40.6
Economic situation	89	13.1
Uncertainty	57	8.4
No social contact with family and friends	55	8.1
Family health status	51	7.5
School life	51	7.5
Childcare	26	3.8
Work family conflict	14	2.1
Family situation	13	1.9
Healthcare	12	1.8
Restrictions	11	1.6
Effects on children	10	1.5
Social impact	7	1
Emotional overwhelm	4	0.6
Ambiguous	3	0.4

### Relation between parental symptoms, family burden, and both parents' and children's worries about the COVID-19 crisis

Parents' GAD-7 scores correlated positively and significantly with parents' worry about the COVID-19 crisis, *r* = 0.32, with children's worry about the COVID-19 crisis, *r* = 0.14, and with family burden, *r* = 0.36 (all *p*s < 0.001), respectively. Parents' GAD-7 scores were also positively and significantly correlated with their worry about another COVID-19 wave, *rb* = 0.25, *p* < 0.001.

**Figure 1:**
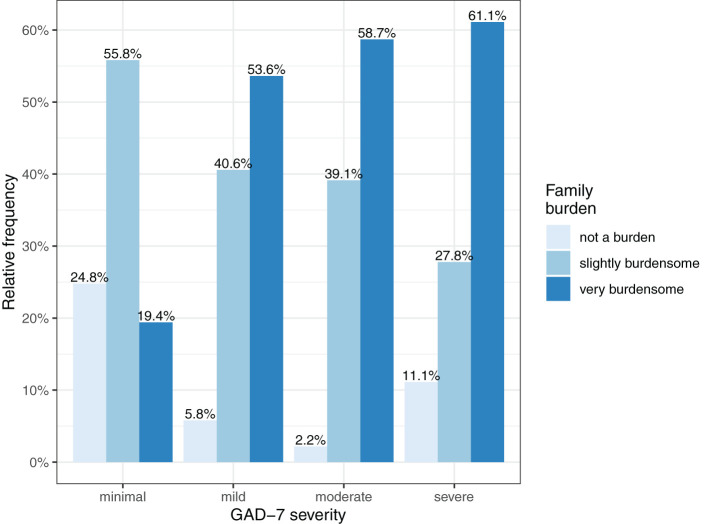
Severity of family burden according to parental GAD-7 symptom severity.

**Figure 2:**
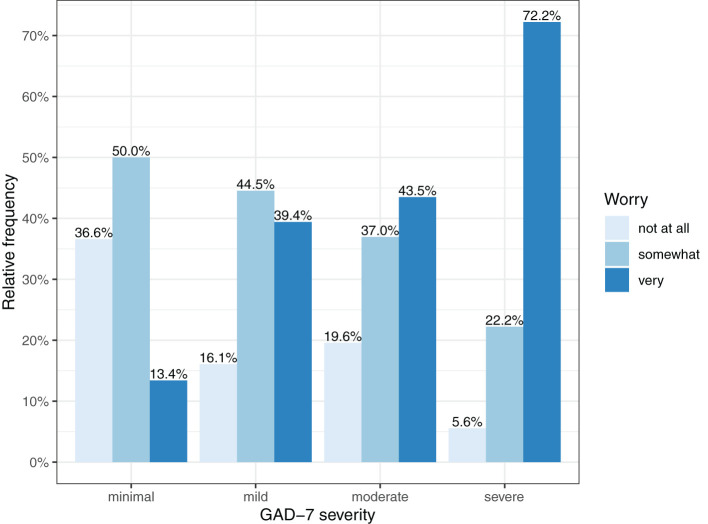
Severity of worry shown for various levels of parental GAD-7 symptoms.

Figures 1, 2 show the severity of family burden and worry about the COVID-19 crisis, subdivided by GAD-7 symptom severity categories. More severe GAD-7 scores were related with more severe family burden and worry. Figure 2 shows that 72.2% of parents with severe GAD-7 scores reported to be very worried about the COVID-19 crisis, while only 13.4% of parents with minimal GAD-7 scores reported to be very worried.

### Children's adverse mental health symptoms and health behaviors as perceived by their parents

**Figure 3:**
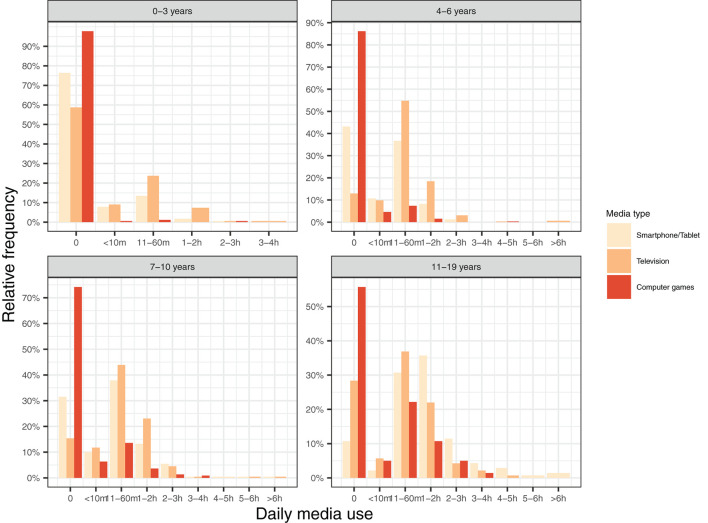
Proportion of children using three types of media for different durations (in min/h) separated by age group.

Media use by age group is shown in Figure 3. Media use increased with age. Among children aged 0–3, about one-third watched TV every day, while more than 20% used a smartphone or a tablet every day. In the age group of 4–6, over 20% of children watched TV for more than 1 h per day, whereas around 10% of children used a smartphone or tablet for more than 1 h per day. Among children aged 7–10, over 25% watched TV more than 1 h per day and over 15% used a smartphone or tablet for more than 1 h per day.

Nearly half (48.1%) of parents reported an increase in their children's media use. Among children aged 11–19 years, 68% of parents reported that media use increased during the pandemic. Furthermore, 13.4% of parents reported an increase in their children's aggression, and 11.7% reported an increase in their children's sleep problems.

### Relationship between parents' generalized anxiety and depression symptoms and their children's symptoms

Logistic regression analyses showed that parents' generalized anxiety (GAD-7) predicted increases in aggression, media use and sleep problems in their children. The results of the logistic regressions are presented in Table 5.

**Table 5:** Results of logistic regressions with parental generalized anxiety (GAD-7) as predictor.

	***B***	***SE***	***z***	***p***	**Odds ratio**	**CI lower**	**CI upper**
**Outcome: increases in aggression in children**							
Intercept	−2.60	0.16	−16.38	<0.001			
Parental generalized anxiety (GAD-7)	0.18	0.02	7.73	<0.001	1.20	1.15	1.26
**Outcome: increases in media use in children**							
Intercept	−0.26	0.09	−2.99	0.003			
Parental generalized anxiety (GAD-7)	0.07	0.02	3.68	<0.001	1.07	1.03	1.11
**Outcome: increases in sleep problems in children**							
Intercept	−2.55	0.15	−17.09	<0.001			
Parental generalized anxiety (GAD-7)	0.14	0.02	5.95	<0.001	1.15	1.09	1.20

CI95 for odds ratio. Outcome **aggression**: R^2^ = 0.10 (Hosmer–Lemeshow), 0.08 (Cox–Snell), 0.14 (Nagelkerke). Model χ^2^(1) = 60.69, p <0.001. Outcome **media use**: R^2^ = 0.01 (Hosmer–Lemeshow), 0.01 (Cox–Snell), 0.02 (Nagelkerke). Model χ^2^(1) = 14.24, p <0.001. Outcome **sleep problems**: R^2^ = 0.05 (Hosmer–Lemeshow), 0.04 (Cox–Snell), 0.07 (Nagelkerke). Model χ^2^(1) = 32.79, p <0.001.

GAD-7 and PHQ-8 correlated highly as mentioned above. Therefore, parental depression (PHQ-8) also predicted increases in aggression, media use and sleep problems in children. Results of these logistic regressions are depicted in Table 6.

**Table 6:** Results of logistic regressions with parental depression (PHQ-8) as predictor.

	***B***	***SE***	***z***	***p***	**Odds ratio**	**CI lower**	**CI upper**
**Outcome: increases in aggression in children**							
Intercept	−2.47	0.15	−16.20	<0.001			
Parental depression (PHQ-8)	0.16	0.02	6.84	<0.001	1.17	1.12	1.22
**Outcome: increases in media use in children**							
Intercept	−0.30	0.09	−3.44	<0.001			
Parental depression (PHQ-8)	0.08	0.02	4.39	<0.001	1.09	1.05	1.13
**Outcome: increases in sleep problems in children**							
Intercept	−2.54	0.15	−17.10	<0.001			
Parental depression (PHQ-8)	0.14	0.02	6.25	<0.001	1.15	1.10	1.20

CI95 for odds ratio. Outcome **aggression**: R^2^ = 0.08 (Hosmer–Lemeshow), 0.06 (Cox–Snell), 0.11 (Nagelkerke). Model χ^2^(1) = 46.47, p <0.001. Outcome **media use**: R^2^ = 0.02 (Hosmer–Lemeshow), 0.03 (Cox–Snell), 0.03 (Nagelkerke). Model χ^2^(1) = 20.87, p <0.001. Outcome **sleep problems**: R^2^ = 0.06 (Hosmer–Lemeshow), 0.04 (Cox–Snell), 0.08 (Nagelkerke). Model χ^2^(1) = 37.07, p <0.001.

## Discussion

The present study is a large-scale telephone survey of parents of children with special needs. A substantial proportion of parents of children with special needs experienced generalized anxiety and depression symptoms during the COVID-19 pandemic, with most also feeling burdened or worried. Parents were in particular worried about a COVID-19 infection. In line with our main hypothesis, these parental symptoms of anxiety and depression were both linked to increased family burden and to their children's adverse mental health symptoms and health behaviors such as increased aggression, media use, and sleep problems. Surprisingly, most children with special needs appeared to cope with the pandemic without expressing significant worry. To gain a better understanding of parents' worry about the COVID-19 crisis, the study also identified important underlying themes of concern.

### Parents' mental health and worries during the COVID-19 crisis

Overall, 7.3% of parents of children with special needs met the criteria for current generalized anxiety disorder, while 7.9% met the criteria for current depression. The majority of parents reported feeling burdened (79.1%) or worried (67.8%) because of the COVID-19 crisis. The main themes of the parents' worry about the COVID-19 crisis were COVID-19 infection (40.6%), economic situation (13.1%), uncertainty (8.4%), lack of social contact with family and friends (8.1%), family health status (7.5%), and school life (7.5%). As expected, parents' generalized anxiety was also associated with increased family burden and worry due to the COVID-19 crisis. The positive association between parents' generalized anxiety and their worry due to COVID-19 in our study also extends the findings of Ren et al. (36), who found that mental and behavioral problems of parents of children with special needs predicted parental state anxiety during the COVID-19 pandemic.

It is alarming that parents of children with special needs in our study had a depression rate of 7.9% (based on a cut-off of 10 on the PHQ-8). In contrast, the prevalence of depression in the region of Vorarlberg in Austria was 4.7% in 2019 according to data from the Austrian Health Interview Survey (ATHIS). It also relied on the PHQ-8 (with the same cut-off criteria) and was conducted as a computer-assisted personal interview. This allows the present results to be meaningfully compared with ATHIS data. Our results indicate that the prevalence of depression among parents of children with special needs in 2020 was 68.1% higher than the prevalence in the general population in 2019 in Vorarlberg.

Several international studies showed increased parental symptoms in the general populations during the COVID-19 pandemic. The prevalence of maternal anxiety and depression increased according to a Canadian longitudinal study (59), while results from the Longitudinal Study of Parents and Children (ALSPAC) in UK showed a huge increase in anxiety, but not depression. Dhiman et al. (60) found the prevalence of depression to be as high as 62.5% among caregivers of children with special needs in India. However, previous studies showed that parents of children with autism spectrum disorder and developmental disabilities had overall higher rates of depression (21, 23, 25). A cross-sectional study in China also showed that parents of children with autism spectrum disorder had more mental health problems than parents of children with an intellectual disability or hearing impairment (61). These results highlight the importance of conducting longitudinal studies in parents of children with special needs while also taking into account different types of special needs (e.g., autism spectrum disorder vs. physical impairment). This will enable researchers to make more specific conclusions about increases in parental symptoms of children with special needs during distressing periods such as the COVID-19 pandemic.

### The relation between parent's symptoms and their children's mental health-related behavior

In line with our main hypothesis, parental symptoms of generalized anxiety and depression during the COVID-19 pandemic were associated with adverse mental health symptoms and health-related behaviors in their children with special needs. Specifically, parents' anxiety and depression symptom intensity was positively associated with their children's aggression, media use, and sleep problems. These results add to the growing literature on the relationship between parent and child psychopathology (38). The positive association between parent and child psychopathology during the COVID-19 pandemic in our study is consistent with previous studies showing elevated levels of depression in parents of children with special needs (21–26). Furthermore, Geweniger et al. (62) found that parents of children with special needs had 2.3 times the odds of reporting mental health problems in their children during the COVID-19 pandemic if the parents screened positive for depression.

Our study shows that aggression, media use and sleep problems in children with special needs are important factors that should be considered by both parents and healthcare professionals. Regarding aggression, there are numerous factors that can contribute to it in children with special needs, including underlying medical conditions, sensory overload, and difficulty communicating their needs or feelings effectively, these factors may be exacerbated when their parents are under stress and pressure. Aggression can lead to increased social isolation, academic difficulties, and emotional distress, creating a vicious cycle. Regarding media use, it is clear that generally it can provide a means of staying informed about the pandemic and connecting with others during periods of social isolation. Excessive media use in children is, however, related to negative health effects on weight and sleep. It is also associated with other risks such as exposure to inaccurate, inappropriate, or unsafe content and contacts; and compromised privacy and confidentiality (63). Finally, studies have found a consistent link between parental depression and sleep problems in children (64–66). Parental depression, children's increased media use, aggression and social isolation may have all contributed to sleep problems in children. Sleep is critical to overall health (67) and sleep problems may lead to the development of various mental disorders (68) and to a range of negative health outcomes later in life, including obesity, diabetes, and cardiovascular disease (69–72). Pediatricians should therefore be vigilant in identifying sleep problems in young children, as these problems can have far-reaching negative health consequences.

Surprisingly, most children with special needs (73.1%) were rated by their parents as being not at all worried about the COVID-19 crisis. This is in line with the study of Ren et al. (36), who found that the correlation between parent and child worry about the COVID-19 crisis was low (*r* = 0.14). Overall, children may have coped with the COVID-19 crisis with less worry than parents, but they may have experienced other symptoms instead such as increased aggression, media use or sleep problems. Furthermore, parents might have difficulties in assessing the degree of their children's worry.

### Strengths and limitations of the present study

Our study has several strengths. First, the main strength of our study is its large sample of almost one thousand parents of children with special needs in Austria, Europe. Our sample is characterized by a variety of educational, economic, and first language characteristics. An exceptionally high response rate of 51.2% distinguishes our study from others and ensures high quality data. Many studies with large samples have been conducted in countries with divergent health care systems, such as China. Therefore, the generalizability of our findings to other western countries is more accurate. Second, our study also has strengths from an ethical perspective. At a time when families were completely isolated, we took the time to contact families personally. Parents were recognized in their needs and were referred to other services when needed. Thus, our study reduced the COVID-19-related burden on the study participants. Third, telephone interviews offer direct personal interaction in real time, while being still cost-effective and time-efficient (50). However, telephone interviews present also several challenges. We faced some logistical challenges in coordinating suitable time slots for telephone interviews with some parents with busy schedules. In a small number of interviews, we encountered some technical problems such as poor call quality, dropped calls or interruptions. Most of these challenges were overcome during the interview, and if not, contingency plans were in place to reschedule interviews if necessary. Finally, although it is plausible that parental mental health symptoms influence the mental health status of their children, the cross-sectional nature of the study does not allow us to make causal statements. It is possible that the association is at least partly bi-directional as the children's symptoms may also affect their parents' wellbeing and anxiety. Furthermore, parents' distress may affect their reports of their children's mental health (73). We also have no pre-pandemic data of our participants, so it is unclear to what extent their mental health conditions changed during the pandemic. Future studies should attempt to distinguish between more homogeneous subsamples that differ in their responses to the COVID-19 pandemic and the lockdowns (74).

### Practical implications

The study also sheds light on the significant burden that caring for a child with special needs can place on families, particularly in times of crisis. Addressing this burden may be key to improving outcomes for both parents and children. This highlights the importance of addressing the needs of the whole family, rather than focusing on individuals, as part of a comprehensive approach to caring for these children. It raises questions about how families with children with special needs can be supported in times of crisis.

The findings of this study have important policy implications. They highlight the importance of considering the unique needs and challenges of families with children with special needs in public health crisis planning and management. Policy and decision makers may need to consider providing additional resources and funding for mental health services and other forms of support for families with special needs children. This includes ensuring that families have (digital) access to parent counseling or peer support groups, especially during periods of social distancing and lockdowns. Families with special needs require additional psychosocial support. Untreated parental mental health problems, such as depression, are associated with impaired therapeutic progress in children (40, 75, 76). Therefore, parental mental health should be routinely assessed to ensure that mental disorders in parents of children with special needs do not remain untreated. Given restraints on resources mental health symptoms may be assessed with health apps (77, 78). Further, as parents of children with special needs experience increased levels of stress, they may not be able to seek help themselves. Service providers and pediatricians should provide information and guidance to at-risk parents in order to help them obtain effective treatment for their children.

The importance of pediatricians in improving the mental health of children with special needs and their parents cannot be overstated. Pediatricians are a good first point of contact for any health-related problems of these children, as they usually have extensive clinical experience in determining whether they require additional health care. Parents usually trust pediatricians and might find it easier to open up to them than to a child psychiatrist or psychologist. Seeking psychosocial help is in most cases still associated with shame, guilt, and stigma (79).

For this reason, good collaboration between pediatricians and psychosocial institutions can provide low-threshold psychosocial support to parents with special needs. Collaboration between pediatricians, child- and adolescent psychiatrists and other specialized therapists (e.g. psychologists, speech-, occupational-, music-, and physical therapists) can also facilitate accurate diagnoses, identification of appropriate psychosocial needs, as well as effective and efficient treatment planning. These factors could also play an important role in improving treatment motivation and adherence. Therapy outcomes in children depend to a large extent on the motivation and ability of parents to participate in their children's treatment. Parents need to be motivated to encourage regular exercise and to keep regular appointments.

### Future research directions

This study provides a snapshot of the potential impact of parental anxiety on children with special needs during the COVID-19 pandemic. Future research could examine specific interventions that could mitigate the negative impact of parental anxiety on child outcomes. This study found a significant relationship between parental generalized anxiety and adverse child mental health outcomes, such as increased media use, aggression, and sleep problems. Future studies could examine potential underlying mechanisms at both the individual and family levels, such as parenting stress, parenting style, (perceived) social support, or coping strategies of all family members. A good starting point could be the themes of parental worry identified in the present study, such as fear of COVID-19 infection, financial burden, social isolation, and general uncertainty. These aspects could be explored quantitatively in future studies and addressed in targeted prevention and intervention strategies.

## Data availability statement

The raw data supporting the conclusions of this article will be made available by the authors, without undue reservation. The additional ATHIS dataset analyzed in this study was obtained from Statistik Austria (http://statistik.gv.at). Requests to access that dataset should be directed to Statistik Austria (info@statistik.gv.at).

## Ethics statement

The requirement of ethical approval was waived by the Ethics Committee of the State of Vorarlberg for the studies involving humans, as ethical approval was not necessary under the legislation of the State of Vorarlberg, Austria. The study was conducted in accordance with local legislation and institutional requirements. Informed consent to participate in this study was obtained via telephone.

## Author contributions

PG: Formal analysis, Visualization, Writing—original draft, Writing—review & editing. KG: Conceptualization, Data curation, Formal analysis, Funding acquisition, Methodology, Project administration, Writing—review & editing. NS: Data curation, Formal analysis, Writing—review & editing. CB: Writing—review & editing. EH: Conceptualization, Writing—review & editing. SB: Conceptualization, Funding acquisition, Investigation, Methodology, Project administration, Resources, Supervision, Writing—review & editing.

## Footnotes

^1^Calculated according to The American Association for Public Opinion Research (54).
